# Foraging and thermally induced phenotypic plasticity interact in the most northerly distributed freshwater fish

**DOI:** 10.1098/rsbl.2024.0636

**Published:** 2025-06-25

**Authors:** Colin E. Adams, Colin Bean, Kevin Parsons

**Affiliations:** ^1^ School of Biodiversity, One Health & Veterinary Medicine, University of Glasgow, Glasgow G12 8QQ, UK; ^2^ NatureScot, Clydebank G81 2NR, UK

**Keywords:** plasticity, climate change, adaptive divergence, morphometrics, Arctic charr

## Abstract

Elevated temperatures from climate change are predicted to be more extreme at higher latitudes. This could require phenotypic plasticity to generate variation that allows organisms to persist in these regions. However, climate change will provide a multifactorial change in environmental cues, making an understanding of how they interact essential for predicting persistence and future evolutionary potential. Here, the impacts of temperature on ecologically relevant phenotypic plasticity (foraging environment) in Arctic charr (*Salvelinus alpinus*) were studied. Eggs and alevins were kept at the same temperature (9°C) and split using a factorial design. This included two temperature treatments (10°C and 14°C) and two treatments representing benthic and pelagic foraging styles. We measured morphology in response to these treatment combinations and found an interaction between foraging and temperature-induced plasticity in body shape that included changes in body depth and the caudal peduncle that could impact swimming ability and fitness. This indicates that thermal conditions may change how plasticity responds to ecological conditions and impact adaptive variation.

## 1. Introduction

Environmental conditions modulate the expression of variation through phenotypic plasticity to impact individual fitness [1,2]. Indeed, plasticity can allow organisms to persist in novel conditions by allowing phenotypes to shift closer to a fitness optimum [3–6]. Conversely, maladaptive plasticity can also occur and shift phenotypes away from fitness peaks [7,8]. These phenomena are becoming increasingly relevant given threats from a range of human-induced changes [1]. Among these threats, climate change is predicted to have broad impacts on biodiversity as global temperatures increase. Besides warmer temperatures, climate change will likely introduce novel effects through ecosystem changes and adjusting community interactions that will alter available niches [1,9–11]. This, in turn, should alter the suite of environmental cues experienced by organisms subject to climate change.

Populations will be at variable risk depending upon their location and biological characteristics. For example, habitats located near the poles are predicted to face greater temperature increases relative to lower latitudes [12–17]. Indeed, conservative estimates project increases for this region of 4°C over the coming century [18]. Additionally, ectotherms are less able to regulate temperature relative to endotherms and are therefore more vulnerable to external temperature change [19–21]. While rising temperatures could result in range shifts, this capacity will, for some species, be restricted due to a combination of geographic factors and dispersal capabilities. This will be particularly true where suitable habitat is fragmented, such as mountain tops, islands or lakes, where populations will be required to adapt or face extinction [21,22]. Therefore, a better understanding of how temperature affects range-restricted ectotherms, especially those from extreme latitudes, will inform the most urgent conservation scenarios [1].

Obligate freshwater fishes living in recently glaciated lakes will face significant pressures from climate change, given that they are ectotherms often with restricted geographical ranges and powers of dispersal [23]. Field studies indicate that elevated temperatures change the timing of spawning, growth rate, bone development and body size [2,17,24–26]. However, field-based studies do not allow such phenotypic shifts to be directly attributed to temperature as there are confounding environmental factors in nature, or observed effects may be explained by selective mortality rather than within-generation phenotypic shifts. Laboratory-based studies can isolate the effects of temperature from other factors that could influence phenotypic variation. This is relevant as climate change will likely include complex changes to entire ecosystems, including prey availability and foraging in fishes [16,27,28]. While morphological variation has been a focus of plasticity research in fish [29–31], these studies have largely focused on diet or foraging effects, with no understanding of how these may interact with other environmental factors.

Among northern fishes, the Arctic charr (*Salvelinus alpinus* (L.)) represents an ideal species to investigate the interaction between temperature and foraging-induced plasticity. This is because Arctic charr are known to display high levels of morphological variation including examples of resource polymorphism [32–37]. For example, there are pelagic, benthic and piscivorous feeding specialist ecomorphs living in sympatry and feeding on zooplankton, macrobenthic invertebrates and fish, respectively, in Loch Rannoch, Scotland [38,39]. In this system, a significant portion of this morphological diversity is attributable to adaptive phenotypic plasticity in response to foraging-induced variation [38,39]. While this loch currently supports multiple ecomorphs, climate change could dramatically alter ecological processes which impact prey availability while also impacting the morphology of the fish directly through temperature-induced plasticity. Therefore, multiple interacting factors including diet and temperature are likely to influence future plasticity and evolution.

To examine the combined effect of temperature and exposure to alternative foraging niches, we conducted an experiment to examine morphological plasticity in response to forecasted future temperatures and its interaction with foraging style. With regard to foraging style, we predicted the induction of morphological variation, in line with previous studies of plasticity in fish [31]. We also hypothesized that temperature would influence morphology directly, with the prediction that temperature and foraging style would interact and cause fish under the warmer temperature regime (facing faster growth) to exhibit a greater magnitude of response to the foraging treatment than under cooler conditions. By studying how temperature interacts with other ecological variables, we hoped to gain insights into the developmental implications of climate change.

## 2. Methods

### (a) Population source and creation of families

For rearing experiments, families of Arctic charr were created from the pelagic ecomorph of Loch Rannoch, Scotland. This population is at the extreme southern range at low altitude for Arctic charr [40]. Fish were captured in November 2014 using gill nets with bar-to-bar mesh sizes of 30−35mm. Females (*n* = 12) were strip spawned on site and eggs fertilized with milt from males (*n* = 12) creating 12 independent families. The resulting embryos were incubated in a re-circulating system with water maintained at 9°C, a temperature of concern for peripheral populations (such as Loch Rannoch) and reflective of a warming scenario [41,42]. This was chosen, as such warmed conditions are predicted to be accompanied by wider fluctuations in weather, including extreme temperatures.

### (b) Foraging and temperature manipulation experiment

Individuals were kept in family groups until a sufficient size to be tagged using visible implant elastomers (mean weight of fish: 0.552 g) to allow re-identification of families. To enable this, fish at six months post-fertilization were anaesthetized using a benzocaine solution (5 ml benzocaine per 1 l of water) and marked laterally on either the right or left side using a 0.3 ml syringe/29 g needle, using a combination drawn from five colours of elastomer (Northwest Marine Technology Inc., Shaw Island, WA, USA). Following a recovery period of 28 days to reduce the chances of tag loss, families were equally divided into four treatment groups comprising two temperature and two diet combinations as follows: those exposed to a benthic diet at 10°C or 14°C, and those exposed to a pelagic diet at 10°C or 14°C. Three replicate groups were used for each treatment type with each containing 20(±3) individuals with families split equally between treatments.

Temperatures chosen mimicked the IPCC estimate of a 4°C increase for Arctic regions over the next century [18], but also a situation of extreme summer conditions (i.e. 14°C). Such a high temperature can cause delays in ovulation for charr [43], but reproduction does not cease. Foraging treatments mimicked the foraging styles of the benthic and pelagic foraging ecomorphs [35,39]. Since nutritional variation can influence fish morphology [44], diet composition was kept consistent between experimental groups with only the mechanical demands of feeding altered between treatments. The quantity of food provided was equalized across both temperature conditions. This followed the protocol of previous experiments on charr that have induced plastic responses in morphology [45,46]. Both diets consisted of dry commercial fish pellets (Trout pellets, Start 1P 25; EWOS, Surrey, B.C., Canada), sheep liver and bloodworms (chironomid larvae) fed in a daily rotation. Pellet particle size was larger for benthic treatments and dispensed using a turkey baster at the bottom of the tank requiring a biting mode of feeding to imitate the foraging habitat of the benthic ecomorph of Loch Rannoch. Additionally, bloodworm and liver composition (approx. 2 : 1 ratio) was frozen onto pieces of PVC pipe from which the ‘benthic’ treatment fish had to pry the food. In contrast, fish exposed to the pelagic treatment were fed smaller pellet particles dropped into the water column and the bloodworm and liver mixture (cut into fragments) was delivered by Pasteur pipette also into the water column to induce a suction mode of feeding.

Given that temperature influences fish growth, the growth potential among the treatment groups was standardized to minimize allometric effects on shape. Therefore, as previous studies have confirmed that temperature over this range has a linear effect on growth, temperature was measured daily to calculate degree-days (also referred to as thermal time [47]) to use as a metric for potential growth and standardized across temperature treatments to limit allometric effects [48–50]. Once fish had reached approximately 4000 (+/–50) degree days (or 162 and 119 actual days elapsed for cold and warm treatments, respectively), they were euthanized with an overdose of benzocaine (10 ml of benzocaine per 1l of water) following UK Home Office schedule 1 guidelines. This meant that plasticity experiments ended when both temperature treatments had reached an equivalent stage of development.

### (c) Geometric morphometric and statistical analysis

**Figure 1.**
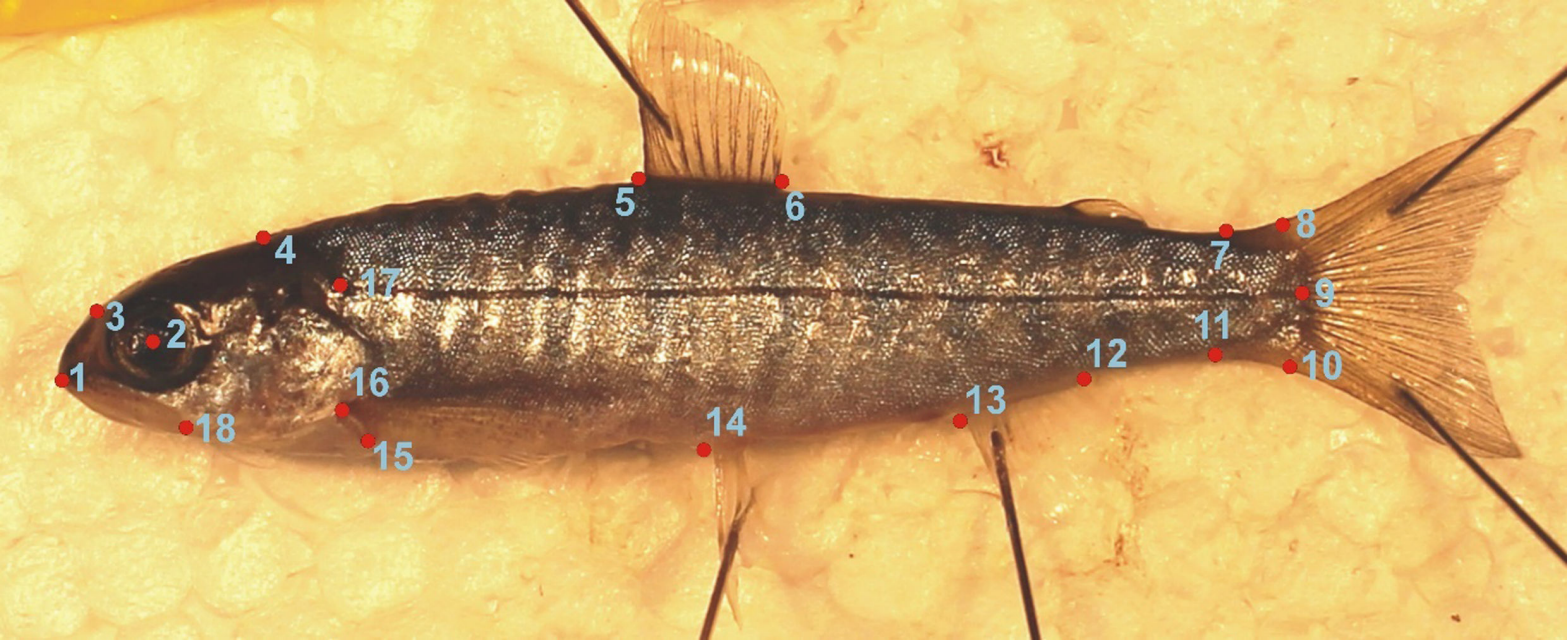
Landmark placement for 18 homologous landmarks. Landmarks were placed as follows: 1, placed on the anterior point of the premaxilla; 2, placed in the centre of the eye; 3, placed on the point of bend on the frontal bone; 4, placed on the dorsal surface of the skull at the connection with the body in line with the gill operculum; 5 and 6, placed at the anterior and posterior insertions of the dorsal fin, respectively; 7, placed on the dorsal of the narrowest part of the caudal peduncle; 8, 9 and 10, placed at the dorsal, centre and ventral insertions of the caudal fin; 11, placed on the ventral of the narrowest part of the caudal peduncle; 12 and 13, placed at the posterior and anterior of the anal fin insertion, respectively; 14, placed at the connection with the left pelvic fin; 15 and 16, placed at the ventral and dorsal insertion with the pectoral fins; 17, placed at the most anterior point of the lateral line and 18, placed at the ventral point of the gill operculum cover.

To assess variation in shape as a result of temperature and foraging treatments, a geometric morphometric analysis was performed. From digital photos landmarks (figure 1) were collected using tpsDig2 (freely available at: http://life.bio.sunysb.edu/morph/index.html). To perform statistical analysis, we used the geomorph package [51] using the R programming language (v. 4.4.153) [52].

For morphometrics, fish were photographed on their left side along with a scale, using a copy stand mounted with a Canon EOS 1100D (Canon Inc., Tokyo, Japan). A total of 18 homologous landmarks were then marked on each image. Landmarks were chosen on the basis of previous studies on Arctic charr to capture ecologically relevant differences in shape [45].

Following their collection, landmark coordinates were adjusted using a general Procrustes analysis. This procedure positioned each specimen to a common centroid, scaled all specimens to a shared unit size, and rotated each specimen to a shared orientation therefore minimizing any squared differences between corresponding landmarks [53].

Statistical models aimed to determine the relative impacts of temperature, foraging style, family and their interaction on morphology. We applied models based on residual randomization in permutation procedures (RRPP) using *ProcD.lm* in geomorph. This non-parametric method is especially useful for high-dimensional data where the number of variables exceeds the number of observations [54]. To account for allometric effects, we included geometric centroid size as a covariate within this model. Because of our balanced design, emphasis on testing temperature effects, and interest in detecting interactions, we applied RRPP models using ordinary least squares and type I sums of squares in the following configuration:


$$Shape\;∼Temperature{\;}^{*}\;\;Foraging\;Style{\;}^{*}\;\;Family{\;}^{*}\;Centroid\;\;Size$$

Because the impacts of experiments on shape could be localized, we conducted our analysis on all landmarks simultaneously, but also subsets to model the effects on just the head (LMs 1−4,16,18) and body (LMs 5−14). All models were run with 1000 permutations, and ANOVA was performed on each using random distributions of F-statistics to calculate z-scores, r-square and *p*-values.

While the main effects of temperature and foraging style in our RRPP models could indicate evidence of their impacts on shape, the proximate ways they impact shape components needed further steps to investigate. Specifically, when interactions between foraging environment and temperature were found we tested for impacts on the direction and magnitude of shape trajectories. Thus, we compared whether the magnitude and direction of plastic responses to diet differed between temperatures. This trajectory analysis was performed using *trajectory.analysis* within the ‘RRPP’ package [54].

Finally, shape variation in fish has well-understood relationships with ecology and function. Therefore, the shape variation induced by our experiments was visualized using deformation grids to depict the bending energy required to change shape. To convey the effects of temperature and foraging style, this was carried out by reciprocally comparing the consensus configuration of each significant factor in our models using the *plotRefToTarget* function and with a 3× magnification of effects to enhance visualization. To convey the effect of interactions among factors, we also produced plots using the fitted values of our models. Briefly, this involved performing a PCA on fitted landmarks from our trajectory analysis, and then generating deformation grids based on a multivariate regression of landmarks against PC1 and PC2 using the *shape.predictor* function to visualize the mean shape against the maximum and minimum values on each PC using *plotRefToTarget*.

## 3. Results

Our combined foraging and temperature experiment provided over 220 individuals, representing a high level of survival (>90%). Our statistical models demonstrated significant changes to morphology in response to foraging style and its interaction with temperature. Specifically, temperature alone did not impact shape, but did interact with foraging style to influence body shape (similar marginal effects in the whole landmark set) (table 1). There were also interactions impacting body shape from an interaction of, foraging style, family and centroid size (table 1). Trajectory analysis revealed that while magnitudes of plasticity did not differ between temperature/foraging groups, while the direction of trajectories did (r = −0.52, angle = 121.2°, *p* = 0.014).

**Table 1.** Results of RRPP models assessing the impacts of temperature, foraging style (FS), family and centroid size (cs) on head, body and whole fish landmark datasets. For *p-*values, asterisks and bolded text indicate significant effects, while periods represent marginal effects.

head	Df	SS	MS	Rsq	F	Z	Pr(>F)	
temperature	1	0.0001	0.0001	0.0015	0.3265	−1.2860	0.9000	
FS	1	0.0005	0.0005	0.0083	1.8217	1.1483	0.1110	
family	1	0.0003	0.0003	0.0054	1.1796	0.6075	0.2710	
cs	1	0.0000	0.0000	0.0003	0.0603	−3.7199	1.0000	
temperature:FS	1	0.0004	0.0004	0.0062	1.3706	0.7889	0.2360	
temperature:family	1	0.0004	0.0004	0.0073	1.5964	0.9978	0.1760	
FS:family	1	0.0001	0.0001	0.0024	0.5193	−0.5188	0.6910	
temperature:cs	1	0.0001	0.0001	0.0012	0.2530	−1.6089	0.9420	
FS:cs	1	0.0004	0.0004	0.0074	1.6275	1.0835	0.1380	
family:cs	1	0.0002	0.0002	0.0034	0.7367	−0.0369	0.5140	
temperature:FS:family	1	0.0005	0.0005	0.0082	1.7965	1.1454	0.1370	
temperature:FS:cs	1	0.0003	0.0003	0.0049	1.0782	0.4021	0.3430	
temperature:family:cs	1	0.0002	0.0002	0.0043	0.9509	0.3193	0.3900	
FS:family:cs	1	0.0004	0.0004	0.0076	1.6755	1.1010	0.1340	
temperature:FS:family:cs	1	0.0002	0.0002	0.0034	0.7361	0.0006	0.5020	
residuals	204	0.0528	0.0003	0.9284				
total	219	0.0568						

body								
temperature	1	0.0004	0.0004	0.0040	0.9000	0.1336	0.4370	
FS	1	0.0005	0.0005	0.0053	1.2026	0.5649	0.2950	
family	1	0.0009	0.0009	0.0100	2.2594	1.7170	**0.0460**	*
cs	1	0.0000	0.0000	0.0005	0.1142	−3.5474	1.0000	
temperature:FS	1	0.0009	0.0009	0.0100	2.2619	1.6121	**0.0500**	*
temperature:family	1	0.0004	0.0004	0.0039	0.8894	0.0777	0.4720	
FS:family	1	0.0006	0.0006	0.0066	1.4969	0.9769	0.1650	
temperature:cs	1	0.0003	0.0003	0.0032	0.7228	−0.2676	0.6060	
FS:cs	1	0.0011	0.0011	0.0122	2.7665	2.0835	**0.0200**	*
family:cs	1	0.0003	0.0003	0.0029	0.6443	−0.5023	0.6940	
temperature:FS:family	1	0.0009	0.0009	0.0093	2.0987	1.5265	0.0640	.
temperature:FS:cs	1	0.0008	0.0008	0.0083	1.8724	1.3032	0.0940	.
temperature:family:cs	1	0.0004	0.0004	0.0047	1.0564	0.3633	0.3610	
FS:family:cs	1	0.0015	0.0015	0.0159	3.6062	2.5857	**0.0060**	*
temperature:FS:family:cs	1	0.0002	0.0002	0.0017	0.3765	−1.4357	0.9270	
residuals	204	0.0828	0.0004	0.9016				
total	219	0.0918						

whole landmark set								
temperature	1	0.0005	0.0005	0.0029	0.6409	−0.5543	0.7130	
FS	1	0.0010	0.0010	0.0062	1.3914	0.8379	0.2050	
family	1	0.0012	0.0012	0.0076	1.7068	1.3026	0.1000	.
cs	1	0.0001	0.0001	0.0004	0.0880	−5.4000	1.0000	
temperature:FS	1	0.0013	0.0013	0.0084	1.8748	1.3833	0.0820	.
temperature:family	1	0.0009	0.0009	0.0054	1.2172	0.6508	0.2540	
FS:family	1	0.0008	0.0008	0.0047	1.0475	0.4037	0.3530	
temperature:cs	1	0.0004	0.0004	0.0025	0.5534	−0.8135	0.7860	
FS:cs	1	0.0017	0.0017	0.0105	2.3436	1.8394	**0.0380**	*
family:cs	1	0.0005	0.0005	0.0030	0.6780	−0.4262	0.6720	
temperature:FS:family	1	0.0013	0.0013	0.0083	1.8609	1.3624	0.0940	.
temperature:FS:cs	1	0.0011	0.0011	0.0066	1.4735	0.9232	0.1830	
temperature:family:cs	1	0.0008	0.0008	0.0048	1.0732	0.4432	0.3350	
FS:family:cs	1	0.0023	0.0023	0.0143	3.1998	2.3741	**0.0090**	*
temperature:FS:family:cs	1	0.0004	0.0004	0.0026	0.5784	−0.6972	0.7680	
residuals	204	0.1468	0.0007	0.9118				
total	219	0.1610						

**Figure 2.**
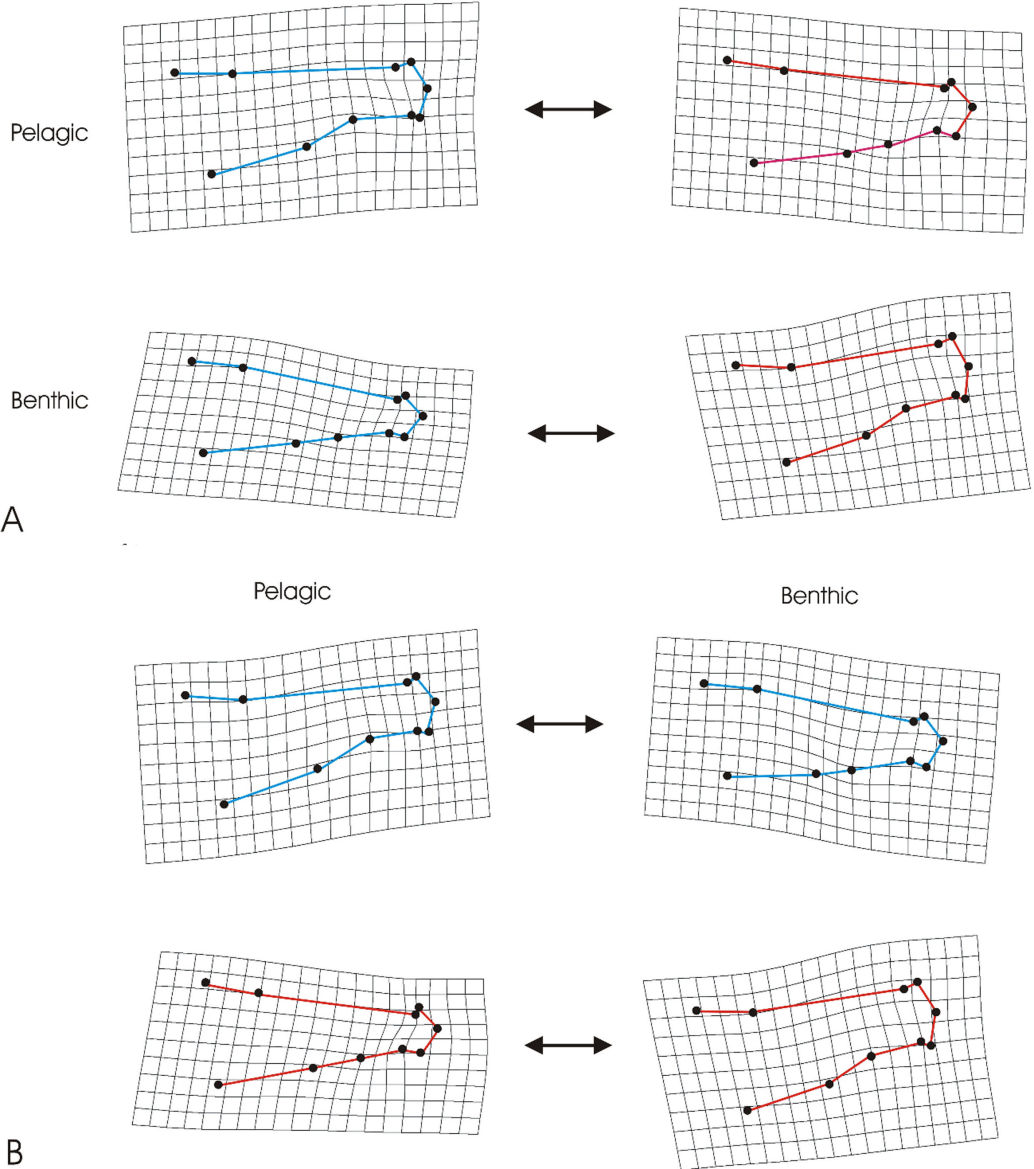
Deformation grids (magnified 3×) representing the differences in shape induced by foraging style (benthic or pelagic) and temperature (10°C or 14°C). Comparisons in panel (A) reflect plasticity induced under temperature treatments but while also being under different foraging style experiments. Here deformation grids are modelled on the basis of temperature-induced differences but with groups separated by foraging treatment. Comparisons in panel (B) reflect shape changes in the same fish as panel (A), but with deformation grids modelled on the basis of foraging treatment, but separated by temperature treatment.

**Figure 3.**
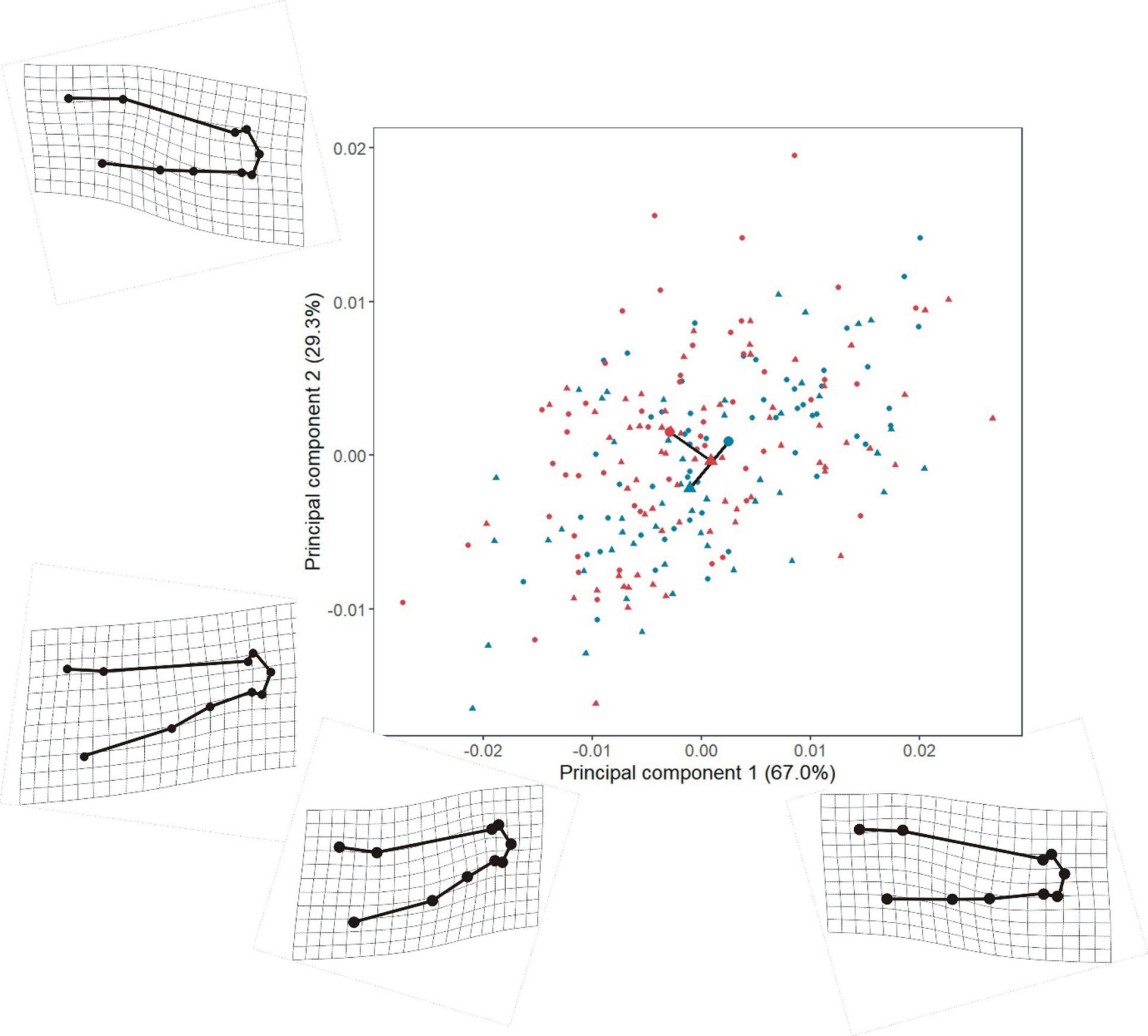
The overall impact of temperature and foraging experiments on body shape in charr as depicted by the main trajectories of shape variation. The values of PC1 and PC2 are derived from fitted values of shape variation from our RRPP model, deformation grids (magnification 5×) depict how body shape changes along each axis. Within the scatterplot red points denote charr raised under 14°C, while blue points denote 10°C treatment charr. Charr provided a benthic foraging style are represented by triangles, while square points represent those under a pelagic treatment. The trajectory lines in the middle of the plot depict mean differences between treatment groups that are perpendicular indicating an interaction between foraging style and temperature.

Deformation grids (figure 2) illustrated plasticity induced through temperature and foraging style (figure 2) and their interaction (figure 3). Under both benthic and pelagic diets, elevated temperatures showed a deepening of the caudal peduncle region (figure 2, panel A). However, an upward curvature was induced in the benthic treatment under 14°C, which differed from the pelagic treatment fish whereby caudal orientation appeared relatively consistent across temperatures. Benthic and pelagic foraging induced differences in body shape, but fish reared at 14°C displayed notably more dramatic changes in shape than those reared at 10°C (figure 2B). Under 14°C, benthic treatments induced a deeper body shape, overall temperature seemed to alter localized regions of shape change across the body.

Treatments, while changing body shape, indicated overlap existed between groups (figure 3). Along both PCs interactions between temperature and foraging style influenced body shape that was either lengthened with a reduction in depth, or shortened with an increase in body depth. More specifically, PC1 indicated some degree of shift in the placement of the caudal fin to a more dorsal or ventral position. PC2 indicated a more marked transition between the body and the beginning of the caudal fin (figure 3).

## 4. Discussion

We found interactions between temperature and foraging-induced plasticity under a scenario that simulated predicted climate warming and environmental stochasticity. While temperature did not alter the degree of morphological plasticity in response to foraging style, there was an effect on the trajectory of plasticity. This suggests that climate change may have a differential effect on fish exploiting different niches through plasticity. If such changes impact fitness and are heritable (as suggested here), the evolutionary potential for populations of fishes is likely to be impacted through direct developmental interactions with temperature and the ecology of the surrounding system. This could be maladaptive and involve heritable variation for how development interprets dietary variation. While this could also include new adaptive variation, it is also possible that such evolution of plasticity could involve counter-gradient mechanisms that maintain fitness benefits of plasticity [55–57].

Plastic responses were limited to the body of charr. This could be due to our experiment starting after charr were of a size capable of handling tagging and thus missing a developmental window for craniofacial effects [2]. Nonetheless, this indicates that elevated summer temperatures could generate some capacity for adaptive responses to different types of prey. However, if limited to the body, these findings suggest that prey consumption will be limited by a relative lack of plasticity in the craniofacial region. This could be mitigated by a shift in prey availability, but developmentally this may also lead to a mismatch, or lack of integration between different anatomical regions under future warmed conditions. Indeed, our previous results from this population indicated an increase in the rate of bone metabolism occurs under elevated temperatures [2]. While this was the general trend, we found that this change was heterogeneous across the anatomy of charr, suggesting a mechanism for how differences in trajectory (but not magnitudes) of plasticity could be occurring here. Charr are known to vary in the degree to which they exhibit plasticity across populations and among ecomorphs [46]. Thus, while we show evidence of interactions, future studies comparing species, populations, and ecomorphs under two factor experimental treatments could be more informative for determining how climate change will impact plasticity.

Given that foraging plasticity is seen as an important factor in the divergence of postglacial fishes its interaction with temperature shown here is of broad importance [1,2,21,31]. Consequently, the phenotypic diversity displayed by Arctic charr could be enhanced by elevated temperatures as they may confer an even greater chance to specialize and diverge based upon different environmental conditions. However, while such morphological changes could be adaptive and functionally relevant, such changes may ultimately incur negative impacts on populations. While morphological variation induced by interactions between temperature and foraging style appear to be ecologically relevant, especially for swimming ability, with elongated and more fusiform shapes advantageous for sustained swimming, they seem disconnected from the craniofacial region. Phenotypic divergence found along the benthic/pelagic habitat axis among Arctic charr ecomorphs and other postglacial fishes follows a pattern whereby the elongation of the caudal region and body is accompanied by an elongated mouth and larger eyes that aid in the sustained swimming and suction feeding required for foraging on zooplankton [29,58]. The interaction between temperature and foraging style found here seems to break this pattern of connection between the head and body, suggesting that effects on function and performance could be negative and warrant further investigation.

Foraging style interactions with family effects suggest a heritable basis. While *p*-values involving family effects did not always meet an alpha value of 0.05, few families were used, and z-scores were relatively high. This suggests an impact on evolutionary potential if this variation is targeted by selection. Indeed, the morphological responses here do not appear to follow the typical bentho-pelagic divergence frequently found in charr, suggesting that interactions with temperature may hamper such divergence. This speaks to the importance of the developmental environment faced by populations [29]. While many salmonid conservation strategies currently focus on spawning habitat restoration, and thus embryo-stage conditions, and post-embryonic developmental conditions are largely neglected. However, the conditions faced by populations at these life stages are likely to be critical for shaping the development of adaptive variation, and ignoring this could seriously threaten populations in a warming world [1,2].

Since climate change is predicted to alter both biotic and abiotic factors, it is important to consider the need for plasticity in such a rapidly changing environment [59]. For example, it has been noted that range shifts caused by climate change have the potential to increase competition for resources as novel species move northwards [59,60]. Similarly, increased temperatures will produce significant alterations in the phenology of many species, thus causing disruption to ‘typical’ food webs [60,61]. Under such a scenario, a change resulting from phenotypic plasticity as a response to foraging could be beneficial. For example, morphological plasticity could allow for persistence in a changing environment when a less plastic species would struggle to adapt. However, the benefits of plasticity are predicated upon the adaptiveness of the traits in question, which is something that cannot be commented on based on our results. However, this raises the possibility of costs for this plasticity [62,63]. While consistent empirical evidence for costs of phenotypic plasticity is lacking, higher levels of early life morphological plasticity could also confer some drawbacks if the environment is not consistent throughout the lifespan of the organism [62–64]. To make assertions regarding whether elevated temperatures induce adaptive, neutral or maladaptive phenotypes requires consideration of the phenotype as a whole.

Morphological plasticity is only one aspect on which climate change will act. Another may be to push Arctic charr towards their upper limit of thermal tolerance, at least at the southern end of their range (such as Scotland, northern England and Wales). It has been demonstrated that eggs and alevins are the most vulnerable life stages to temperature increases [65]. Previous work has shown that juvenile Arctic charr acclimated to higher temperatures have a higher thermal tolerance, suggesting that UK populations may be more resilient to elevated temperatures than those in northern latitudes [66]. It is also possible that heatwaves and extreme weather events could, especially in more shallow lakes, cause Arctic charr to be pushed passed their critical maximum temperature [41]. As stated, charr are relatively range-restricted and therefore climate change could see these fish under increasing selection pressure as surface water temperatures increase in line with air temperatures [67,68]. Interestingly, Arctic charr have a temperature preference which is lower than that predicted for optimal growth and therefore in warming waters there could be increased growth efficiency which may offset some detrimental consequences of higher temperatures [69]. Generally, Arctic charr have adapted to inhabit colder regions, and therefore, any warming will undoubtedly cause stress that will threaten the persistence of populations at the southern end of their range [16,41,42].

## 5. Conclusions

By recognizing that multiple factors induce plasticity, this study has suggested new inferences about organismal responses to climate change. However, there will likely be no generalizable pattern of elevated summer temperatures on morphological plasticity in Arctic charr let alone in similar polymorphic species. Furthermore, the conditions used in this study are representative of predictions over the coming century [41]. There is likely to be additional variability missed by this approach such as the future dynamics of lake stratification and possibly shift in spawning time and location that affect the conditions face by young charr. Indeed, complexity is reflected within charr as levels of plasticity can differ between ecomorphs within the same lake and between lakes [46]. This necessitates not only that similar studies are conducted on multiple populations of interest but also that the adaptive value of these phenotypes is tested [2]. However, given that current conservation practices focus on measuring demographic shifts, usually of species rather than ecomorphs, the study of how environmental change will impact phenotypes is currently underappreciated [1]. This experiment simulated conditions of increased summer temperatures and shows that this scenario alters the scope for morphological responses to foraging style. Given that much current adaptive diversity has evolved under a cooler climate, it is prescient to consider how such variation will fare under the change of environmental conditions predicted for the coming century.

## Data Availability

Landmark data files, and categorical variables with R code from this manuscript are available from the Dryad Digital Repository [70].
